# Unconditional Perseveration of the Short-Term Mental Set in Chunk Decomposition

**DOI:** 10.3389/fpsyg.2018.02568

**Published:** 2018-12-11

**Authors:** Furong Huang, Shuang Tang, Zhujing Hu

**Affiliations:** School of Psychology, Jiangxi Normal University, Nanchang, China

**Keywords:** mental set, chunk decomposition, attentional bias, procedural learning, Chinese characters, cognitive inflexibility

## Abstract

A mental set generally refers to the brain’s tendency to stick with the most familiar solution to a problem and stubbornly ignore alternatives. This tendency is likely driven by previous knowledge (the long-term mental set) or is a temporary by-product of procedural learning (the short-term mental set). A similar problem situation is considered the factor required for perseveration of the long-term mental set, which may not be essential for the short-term mental set. To reveal the boundary conditions for perseveration of the short-term mental set, this study adopted a Chinese character decomposition task. Participants were asked to perform a practice problem that could be solved by a familiar loose chunk decomposition (loose solution) followed by a test problem, or they were asked to repeatedly perform 5–8practice problems followed by a test problem; the former is the base-set condition, and the latter is the enhanced-set condition. In Experiment 1, the test problem situation appeared to be similar to the practice problem and could be solved using the reinforced loose solution and also an unfamiliar tight chunk decomposition (tight solution) (a 2-solution problem). In Experiment 2, the test problem situation differed from the practice problem and could only be solved using an unfamiliar tight solution (a 1-solution problem). The results showed that, when comparing the enhanced-set and base-set conditions, both the accuracy rate and the response times for solving the test problem with a tight solution were worse in Experiment 1, whereas the response times were worse in Experiment 2. We concluded that perseveration of the short-term mental set was independent of the similarity between problem situations and discuss the differences in perseveration between two types of fixation.

## Introduction

A mental set is also known as the Einstellung effect, which represents a form of rigidity in which an individual behaves or believes in a certain manner. In the field of psychology, this effect has typically been examined in the process of problem solving and specifically refers to the brain’s tendency to stick with the most familiar solution and to stubbornly ignore alternatives (Schultz and Searleman, 2002). Both prior knowledge and a similar problem situation were considered the factors required to induce an attentional bias toward the familiar solution (Lovett and Anderson, 1996). In addition, the mental set is also likely formed and strengthened by repeatedly practicing a particular solution in a short time and can be interpreted as a temporary by-product of procedural learning (Ohlsson, 1992; Ollinger et al., 2008). However, whether a similar problem situation is an essential factor for perseveration of the short-term mental set remains largely unknown.

The mental set is likely driven by previous knowledge, particularly expertise in a domain (Wiley, 1998; Ricks et al., 2007; Ellis and Reingold, 2014), which can be defined as the long-term mental set. This mental set always occurs when people are confronted with a problem situation that is similar to previously experienced problem situations. Previously acquired knowledge likely helps problem solvers to understand, interpret and solve problems quickly and also likely has a negative impact. For example, most errors that doctors make are not connected to their inadequate medical knowledge but rather to the tendency to form opinions quickly based on previous experience. Once the initial diagnosis is formed, it guides doctors in the search for supporting evidence, which in turn introduces a risk of missing important aspects unrelated to the initial diagnosis.

In a laboratory experiment, chess players were required to find a checkmate position with the fewest number of moves. If players were given a 2-solution problem that had two possible solutions, a familiar solution that took five moves and a less familiar solution that took three moves (the optimal solution), then most of the players selected the familiar but non-optimal solution and failed to notice the shorter solution (Bilalić et al., 2008). Eye tracking technology revealed that the cognitive mechanism underlying this phenomenon was attentional bias, where previous knowledge likely directs attention toward relevant information and away from irrelevant information. Accordingly, players rapidly fixated on the target region that was associated with the familiar but longer solution (i.e., checkmate in five moves) and spent more time looking at these squares rather than those relevant to the shortest solution (i.e., checkmate in three moves), even when they reported that they were searching for alternative solutions in an open-minded manner (Bilalić et al., 2008, 2010; Sheridan and Reingold, 2013). Thus, the search for a solution became self-fulfilling as the familiar solution was consistent with previously acquired knowledge and was more likely to be utilized (Bilalić et al., 2008, 2010; citealpBR1). If a problem situation is different from previous experiences, then no cues will elicit retrieval of previously acquired knowledge and no attentional bias will occur.

In addition, the mental set is also likely strengthened by repeated practice in a short time and can be interpreted as a temporary by-product of procedural learning (Ohlsson, 1992). One of the most famous examples is the so-called water jar problem, which was originally developed by Luchins (Luchins, 1942; Luchins and Luchins, 1969). Participants are presented with three jars (A, B, and C), each of which holds a certain amount of water. The goal is to determine how the jars can be used to obtain a designated amount of water. A series of practice problems can only be solved using a complicated strategy (e.g., A – B – 2C), which participants learn quickly. Subsequently, the participants are provided a test problem (called the 2-solution problem) that could be solved using either the complicated strategy or a much easier strategy (e.g., A – C). Typically, most participants continue to use the complicated strategy instead of the simple strategy. In this case, fixation is induced by repeatedly reinforcing a small number of similar problems in people who have never experienced the task before, which can be defined as the short-term mental set.

In previous studies, the short-term mental effect has been demonstrated in both the laboratory and real-life settings using a range of different problem-solving tasks (Schultz and Searleman, 2002). However, the neurocognitive mechanism underlying this effect and its boundary conditions remain largely unknown. One possibility is that the reinforced solution gradually realizes mechanization, which likely becomes automatically activated during the next problem when the problem situation is similar to the former practice problems. Accordingly, problem solvers progressively require less time to solve problems with a reinforced solution but also experience greater difficulties in searching for alternative solutions (Neroni et al., 2017). Meanwhile, mechanization of a particular solution likely implies that people’s brains lost flexibility to manage novel stimuli or tasks. Therefore, although the next problem situation was different from the former practice problems, negative influences of the short-term mental set likely remained. More generally, regardless of whether the next problem is similar to the former practice problems, problem solving will be hindered when people try to use alternative solutions rather than the reinforced solution.

To reveal the boundary conditions of perseveration of the short-term mental set, a chunk decomposition task was adopted in this study. As a possible means to solve insight problems, chunk decomposition refers to decomposing familiar patterns into their components such that they can be regrouped in a different and meaningful manner (Knoblich et al., 1999). Based on whether the components of the chunks to be decomposed are themselves meaningful perceptual patterns, chunk decomposition can be divided into loose and tight levels. Decomposing the numeral “VI” into “V” and “I” is an example of loose chunk decomposition, and decomposing ‘X’ into “/” and “∖” is an example of tight chunk decomposition because ‘VI’ is composed of meaningful small chunks (‘V’ and ‘I’), whereas ‘X’ is composed of meaningless small chunks (“/” and “∖”) (Knoblich et al., 1999). Generally, participants are more familiar with loose chunk decomposition rather than tight chunk decomposition due to previous knowledge about chunks (Knoblich et al., 1999; Wu et al., 2013; Huang et al., 2015), but the latter strategy is critical to solving insight problems. Moreover, previous studies have demonstrated that performance in solving mathematical problems with loose chunk decomposition (a loose solution) was improved by repeated practice in the set (Knoblich et al., 2001; Chi and Snyder, 2011), i.e., the short-term mental set of chunk decomposition was formed and strengthened by intense practice. After repeatedly solving 5∼8 practice mathematical problems using a loose solution, participants were asked to solve a test mathematical problem, which was different from the practice problem and could only be solved by tight chunk decomposition (a tight solution), in the experimental condition; or else participants were asked to perform a test mathematical problem after repeatedly solving several anagrams in the control condition (Ollinger et al., 2008). Results showed no significant difference in the performance of the test problem between two conditions. Researchers believe that the short-term mental set did not perseverate in the test problem since it was insightful (Ollinger et al., 2008) and different from the practice problem situation. However, another possibility is that perseveration of the short-term mental set was independent on the problem situation similarity, and was happened in both the experimental condition and the control condition; or the short-term mental set likely perseverate in a totally different problem situation.

To further reveal the boundary condition of the short-term mental set, we selectively adopted the design of Ollinger et al. (2008) in this study. Participants were asked to repeatedly perform 5–8 practice problems that could be solved using a loose solution, followed by a test problem, or they were asked to perform a single practice problem followed by a test problem; the former is the enhanced-set condition, and the latter is the base-set condition. In Experiment 1, the test problem situation appeared to be similar to the practice problem and could be solved by the reinforced loose solution and also an unfamiliar tight solution (a 2-solution problem). In Experiment 2, the test problem situation was different from the practice problem and could only be solved by an unfamiliar tight solution (a 1-solution problem). By comparing the success probability and response time of solving the test problem with an unfamiliar tight solution between the enhanced- and base-set conditions, the influences of the short-term mental set on the unfamiliar tight solution were revealed, allowing examination of whether perseveration of the short-term mental set was independent of the situation similarity between the practice problems and the test problem.

We assumed that the short-term mental set would be formed and strengthened after repeatedly solving several similar practice problems using the loose solution and would negatively influence solving of the test problem with an unfamiliar tight solution. The accuracy rates and response times associated with the tight solution for the test problem would be worse in the enhanced-set condition versus the base-set condition regardless of whether the test problem situation was similar to the practice problems.

## Experiment 1

### Methods

#### Participants

Thirty-two paid participants (18 males between the ages of 18 and 22 years; mean age 20.11 ± 1.31 years) recruited from the Jiangxi Normal University participated in the task as paid volunteers. They were all native Chinese speakers and had normal or corrected-to-normal vision. Before the experiment, all participants signed the informed consent form approved by the institutional review board of the Jiangxi Normal University.

#### Tasks, Design, and Procedure

**FIGURE 1:**
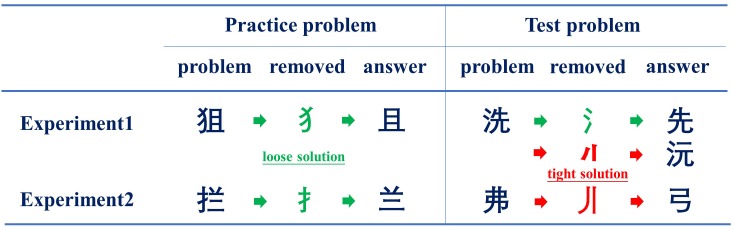
Example of the Chinese character decomposition task in this study.

This study adopted a Chinese character decomposition task in which participants were asked to decompose and remove any part (radicals or strokes) of a character to produce another legal character (Zhang et al., 2015; Tang et al., 2016; Wu et al., 2017). Chinese characters are composed of radicals (sub-chunks that may convey phonetic or semantic information of the character and can be used as an independent unit), which are composed of strokes (basic elements that do not carry any meaning). Character decomposition can occur at either the radical or stroke level, such as decomposition of the character 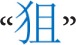 into 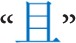 by removing the radical 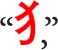 decomposing the character 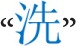 into 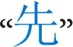 by removing the radical 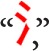 or decomposing the character 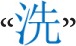 into 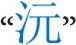 by removing the stroke 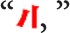 (see Figure 1). According to the mental representation change hypothesis, chunk decomposition can be divided into loose and tight levels depending on whether the components of the chunks to be decomposed are themselves meaningful perceptual patterns (Knoblich et al., 1999). Thus, decomposing Chinese characters by removing a radical was considered a loose solution, whereas removing a stroke was considered a tight solution (Luo and Knoblich, 2007; Luo et al., 2008). In the experiment, the participants were asked to decompose a given character to generate another legal character by removing a radical or a stroke, and no cue toward a loose or tight solution was provided.

Two conditions were created in this study, namely, the base-set and enhanced-set conditions, and their presentation sequences were random. In the base-set condition, the participants were asked to perform a practice problem that could be solved only by a loose solution (decompose and remove radicals), followed by a test problem that could be solved by a loose solution and also a tight solution (decompose and remove strokes). In the enhanced-set condition, the participants were asked to continuously perform 5∼8 similar practice problems, followed by one test problem; the range was designed to prevent participants from anticipating. In both conditions, the test problem situation was similar to the practice problems in which the character to be decomposed had a radical element that was closely associated with the loose solution. In total, 24 practice problems and 24 test problems were included in the base-set condition, and 156 practice problems and 24 test problems were included in the enhanced-set condition. Each problem was a Chinese character that was highly familiar to the participants, who were native Chinese speakers.

**FIGURE 2:**
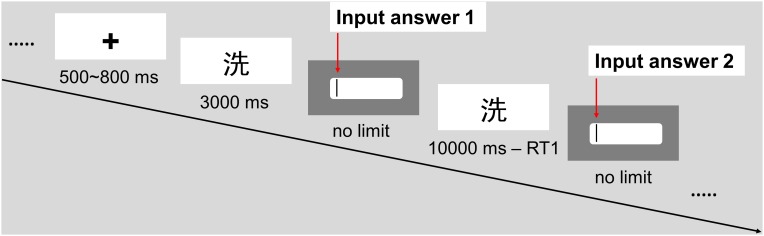
Example of the experimental trial timeline in Experiment 1.

The time course of each trial is shown below (see Figure 2). After a period of 500∼800 ms that was designed to reduce expectation, the character to be decomposed appeared in the center of the screen for up to 3,000 ms. During this period, the participants were instructed to consider the answers one by one and to press a response key with the right index finger as soon as they determined an answer. Then, an input box appeared on the screen, and the participants were given an unlimited period of time to enter their answers using a keyboard and then press the “Enter” key to complete the task. Subsequently, the same character again appeared in the center of the screen for up to 10,000 ms minus the reaction time for the first encounter, and the participants were given an unlimited amount of time to enter their answers using a keyboard, or the participants could press the “Space” key to end the trial if they believed that no other answer was possible. Thus, both the character to be decomposed and the answer input box appeared twice since two answers were required for the test problem, and the same procedure was applied to the practice problem for coherence. After the participants finished a practice problem and a test problem or 5∼8 practice problems and a test problem in the set, a 3∼5-s interval was included as a break. The random length was designed to reduce the impact of expectation and preparation.

### Results

To demonstrate the influences of the short-term mental set on chunk decomposition, we compared the response times and accuracy rates of the loose solution for both the practice and test problems and the tight solution for the test problem between the enhanced-set condition and the base-set condition.

**FIGURE 3:**
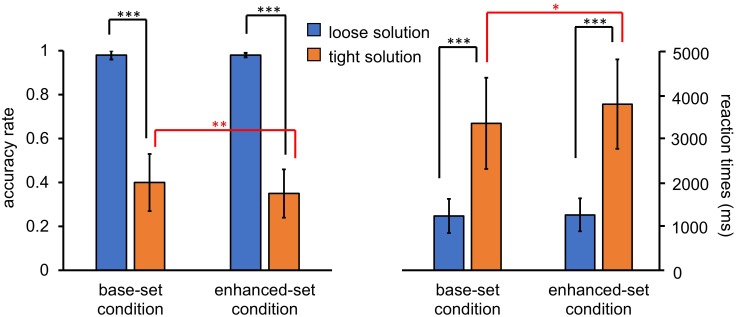
The panel shows the mean accuracy rate and the mean response times for loose and tight solutions for character decomposition in both the base-set and enhanced-set conditions in Experiment 1. Error bars represent the 95% confidence interval. The asterisks indicate significant differences between conditions (^∗^*p* < 0.05, ^∗∗^*p* < 0.01, ^∗∗∗^*p* < 0.001).

For the accuracy rate, a 2 (condition: base-set, enhanced-set) × 2 (solution: loose, tight) repeated-measures analysis of variance (ANOVA) revealed significant effects of the condition [*F*(1,31) = 6.58, *p* = 0.015, η^2^ = 0.18], the solution [*F*(1,31) = 940.16, *p* < 0.001, η^2^ = 0.97], and the interaction effect [*F*(1,31) = 11.00, *p* = 0.002, η^2^ = 0.26]. The participants achieved fewer correct responses for the tight solution in the test task in the enhanced-set condition than in the base-set condition [*t*(31) = 9.42, *p* = 0.004], but no significant differences emerged between the enhanced-set condition and the base-set condition for the loose solution [*t*(31) = 0.24, *p* = 0.63] (Figure 3).

For the mean response times, a 2 (condition: base-set, enhanced-set) × 2 (solution: loose, tight) repeated-measures ANOVA showed significant effects of the condition [*F*(1,31) = 7.75, *p* = 0.009, η^2^ = 0.20], the solution [*F*(1,31) = 203.25, *p* < 0.001, η^2^ = 0.87] and the interaction effect [*F*(1,31) = 5.67, *p* = 0.024, η^2^ = 0.16]. The reaction times of the tight-level solution for the test task were longer in the enhanced-set condition than those in the base-set condition [*t*(31) = 6.87, *p* = 0.013], but no difference in response time for the loose solution for both tasks was found in either condition [*t*(31) = 0.74, *p* = 0.40] (Figure 3).

## Experiment 2

### Methods

#### Participants

Twenty-eight participants (20 males between the ages of 18 and 22 years; mean age 19.93 ± 1.36 years) recruited from the Jiangxi Normal University participated in the task as paid volunteers. They were all native Chinese speakers and had normal or corrected-to-normal vision. Before the experiment, all participants signed the informed consent forms approved by the institutional review board of the Jiangxi Normal University.

#### Tasks, Design, and Procedure

The tasks and design were similar to those in Experiment 1, except that the test problem was different from the practice problems since the character to be decomposed did not have a radical element that was closely associated with the loose solution and could only be solved by the tight solution. Thus, in both conditions, the practice problem could be solved by a loose solution (decompose and remove radicals), whereas the test problem could be solved by a tight solution (decompose and remove strokes). For example, the participants were asked to decompose the character 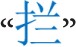 into 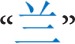 by removing the radical 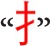 (loose solution) in the practice problems, and to decompose the character 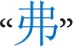 into 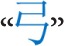 by removing the stroke 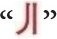 (tight solution) in the test problem (see Figure 1 for examples).

**FIGURE 4:**
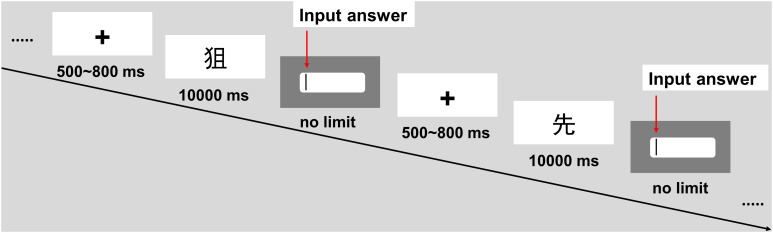
Example of the experimental trial timeline in Experiment 2.

The time course for each trial was as follows (see Figure 4). After 500∼800 ms, the character to be decomposed appeared in the center of the screen for up to 10,000 ms. During this period, the participants were asked to press a response key with the right index finger as soon as they determined an answer. Subsequently, an input box appeared on the screen, and the participants were given an unlimited amount of time to enter their answers using a keyboard and press the “Enter” key to complete the task. After the participants finished a practice problem and a test problem or 5∼8 practice problems and a test problem in the set, a 3∼5-s interval was provided as a break.

### Results

To demonstrate the influences of the short-term mental set on chunk decomposition, we compared the response times and accuracy rates of the loose solution for all practice problems and the tight solution for the test problem between the enhanced-set condition and the base-set condition.

**FIGURE 5:**
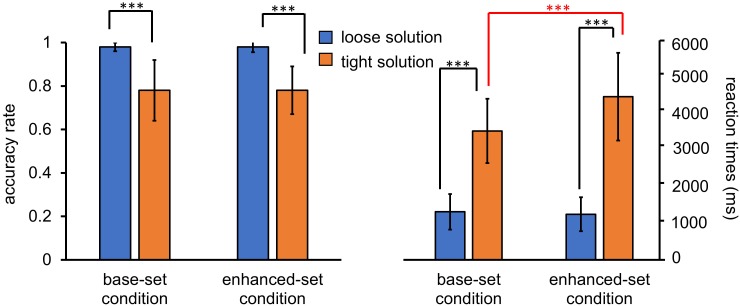
The panel shows the mean accuracy rate and the mean response times for the practice and test problems in both the base-set and enhanced-set conditions in Experiment 2. Error bars represent the 95% confidence interval. The asterisks indicate significant differences between conditions (^∗∗∗^*p* < 0.001).

For the accuracy rate, a 2 (condition: base-set, enhanced-set) × 2 (solution: loose, tight) repeated-measures ANOVA revealed significant effects of the solution [*F*(1,27) = 107.41, *p* < 0.001, η^2^ = 0.80], indicating that the participants had fewer correct responses for the tight solution versus the loose solution, whereas the main effects of the condition [*F*(1,27) = 0.02, *p* = 0.89, η^2^ = 0.001] and the interaction effect [*F*(1,27) = 0.06, *p* = 0.81, η^2^ = 0.002] were not significant (Figure 5).

For the mean response times, a 2 (condition: base-set, enhanced-set) × 2 (solution: loose, tight) repeated-measures ANOVA showed the significant effects of the condition [*F*(1,27) = 16.12, *p* < 0.001, η^2^ = 0.37], the solution [*F*(1,27) = 371.25, *p* < 0.001, η^2^ = 0.93] and the interaction effect [*F*(1,27) = 29.50, *p* < 0.001, η^2^ = 0.52]. The reaction times of the tight solution were longer in the enhanced-set condition than those in the base-set condition [*t*(27) = 23.85, *p* < 0.001], but no difference in response time for the loose solution was found between the two conditions [*t*(27) = 1.18, *p* = 0.29] (Figure 5).

## Discussion

To reveal the boundary conditions of perseveration of the short-term mental set, this study adopted a Chinese character decomposition task. Participants were asked to perform a practice problem that could be solved by a familiar loose solution followed by a test problem, or they were asked to repeatedly perform 5–8 practice problems followed by a test problem; the former task is the base-set condition, and the latter task is the enhanced-set condition. The test problem situation was similar to the practice problem, which included a character with a radical structure, and could be solved by the reinforced loose solution and also an unfamiliar tight solution (Experiment 1), or the situation was different from the practice problem, which included a character without a radical structure, and could only be solved using an unfamiliar tight solution (Experiment 2). The results showed that the participants’ performance in solving the test problems with the unfamiliar tight solution was worse in the enhanced-set condition than in the base-set condition regardless of whether the test problem situation was similar to the practice problems.

For the 2-solution test problem in both the base- and enhanced-set conditions of Experiment 1, all of the participants selected the loose solution as their first choice even though no cue toward a loose or tight solution was provided in the experimental instructions, and the probability of using the loose solution was much higher than that of using the tight solution. This result was consistent with the chunk decomposition hypothesis that chunk decomposition begins with loose chunks, and that the probability that a chunk will be decomposed is inversely proportional to the tightness of the chunk (Knoblich et al., 1999). The processing tendency toward loose chunk decomposition likely reflected the long-term mental set, which originated from previous knowledge about chunks. In particular, Chinese characters are composed of radicals, which are composed of strokes. Because radicals are meaningful elements and can be viewed as independent units, people likely consider removing radicals as the first choice in the process of chunk decomposition when a radical structure is present in the characters (Luo and Knoblich, 2007; Luo et al., 2008). In other words, previous knowledge biased attention toward the radical structure and the corresponding loose solution, which was likely prioritized first when performing the Chinese characters decomposition task.

Compared with the base-set condition of Experiment 1, the participants had a lower probability of identifying and required more time to search for the tight solution for the test problem in the enhanced-set condition, reflecting the negative influence of the short-term mental set. As a temporary by-product of procedural learning, the short-term mental set was formed and strengthened with repeated practice of a particular solution. The solution that was satisfactory for all of the practice problems resulted in gradual realization of mechanization, which was likely automatically activated in the problem situation that was similar with prior practice problems (Lovett and Anderson, 1996). Accordingly, problem solvers become faster at solving similar consecutive problems (Ollinger et al., 2008). In this study, performance in solving the practice problem did not increase in the enhanced-set condition compared with the base-set condition, likely because of a ceiling effect. More importantly, performance in solving the test problem by the unfamiliar tight solution was decreased in the enhanced-set condition versus the base-set condition. Two possible mechanisms may underlie this phenomenon. First, reinforced practice enhanced the attentional bias toward the loose solution since a radical structure was present in the test problem situation and in the practice problems. Second, a particular solution realizing mechanization indicates that cognitive and neural adaptation occurred, and the participants may have lost the flexibility to shift their attention to search for other solutions.

For the 1-solution test problem in both conditions of Experiment 2, no radical element was present for retrieval of the loose solution, and the loose solution did not interfere with the tight solution. Therefore, the accuracy rate of solving the test problem with an unfamiliar tight solution was relatively high. Compared with the base-set condition, the participants showed poorer performance in solving the test problem by the tight solution after repeatedly solving the practice problems by the loose solution. This result revealed that the short-term mental set persisted in a different problem situation even though no attentional bias toward the radical structure and its corresponding loose solution likely occurred. The only possible explanation is that mechanization of a particular solution decreased cognitive flexibility, which likely increased the switching costs from the practiced problems to a totally different problem. Therefore, perseveration of the short-term mental set was independent of the similarity between the problem situations. Regardless of whether the next problem situation is similar to the previously practiced problems, problem solving will be hindered when people try to explore alternative solutions rather than using the repeatedly reinforced solution.

Although the formation mechanisms of the long-term mental set and the short-term mental set are completely different, these two kinds of fixation likely occur at the same time. In particular, the short-term mental set can be formed and strengthened on the basis of the long-term mental set. As in this study, the short-term mental set of chunk decomposition was formed and strengthened after the participants repeatedly solved several practice problems with the loose solution, which was driven by the long-term mental set originating from previous knowledge about Chinese character chunks. Then, when the next problem situation was similar to the previously practiced problems, influences from both the long-term mental set and also the short-term mental set manifested. The former set likely decreased the accuracy rate of solving the test problem with the tight solution due to an attentional bias toward the familiar loose solution, whereas the latter set likely increased the response times of solving the test problem with the tight solution since cognitive flexibility was lost after a particular process realizing mechanization. Therefore, both the accuracy and the response time in solving the test problem with an alternative solution were worse in the enhanced-set condition than those in the base-set condition (in Experiment 1). If the next problem situation was not similar to the previously practiced situation, then the influence from the short-term mental set leads to cognitive inflexibility, which likely affected performance on the switching task. Consequently, the participants spent considerably more time searching for and executing the solution in the enhanced-set condition versus the base-set condition (in Experiment 2). The different influences of the test problem on performance in the two experiments also demonstrated the differences in perseveration of the long-term mental set and the short-term mental set.

In sum, the short-term mental set that was formed and strengthened by repeated reinforcement of a particular solution to solve a set of similar practice problems not only likely increased the attentional bias toward the familiar solution when the test problem situation was similar to the practice problems but also likely decreased cognitive flexibility and increased the switching costs from the practice problems to a totally different test problem. Perseveration of the short-term mental set was independent of the similarity between problem situations. Therefore, the short-term mental set was different from the long-term mental set since the latter can only be induced when a similar situation activates previous knowledge. This study largely broadens our general understanding of the mental set and not only distinguished two types of mental sets on the basis of the forming processes but also revealed the differences in the necessary conditions for perseveration. In future research, the neurocognitive mechanism underlying the two types of fixation should be further investigated.

## Ethics Statement

This study was carried out in accordance with the recommendations of Norms for human behavior experiments in Jiangxi Normal University with written informed consent from all subjects. All subjects gave written informed consent in accordance with the Declaration of Helsinki. The protocol was approved by the institutional review board of the Jiangxi Normal University.

## Author Contributions

FH and ST designed the experiments. ST collected and analyzed the data. FH, ST, and ZH wrote the manuscript.

## Conflict of Interest Statement

The authors declare that the research was conducted in the absence of any commercial or financial relationships that could be construed as a potential conflict of interest.
